# Child stunting starts in utero: Growth trajectories and determinants in Ugandan infants

**DOI:** 10.1111/mcn.13359

**Published:** 2022-04-29

**Authors:** Grace Namirembe, Shibani Ghosh, Lynne M. Ausman, Robin Shrestha, Sonia Zaharia, Bernard Bashaasha, Nassul Kabunga, Edgar Agaba, Julieta Mezzano, Patrick Webb

**Affiliations:** ^1^ Feed the Future Innovation Lab, Friedman School of Nutrition Science and Policy Tufts University Boston Massachusetts USA; ^2^ Feed the Future Innovation Lab for Nutrition Boston Massachusetts USA; ^3^ Department of Agribusiness and Natural Resource Economics Makerere University Kampala Uganda

**Keywords:** child growth, group‐based trajectories, growth trajectories, stunting, wasting

## Abstract

Childhood stunting remains a public health burden worldwide. Although many studies have examined early life and in‐utero risk factors; most have been observational and have used analytic techniques that make inferences limited to population means, thereby obscuring important within‐group variations. This study addressed that important gap. Using data from a birth cohort of Ugandan infants (*n* = 4528), we applied group‐based trajectory modelling to assess diverse patterns of growth among children from birth to 1‐year old. A multinomial regression model was conducted to understand the relationship between risk factors and observed patterns across groups. We found that the onset of stunting occurred before birth and followed four distinct growth patterns: chronically stunted (Group 1), recovery (Group 2), borderline stunted (Group 3) and normal (Group 4). The average length‐for‐age *z*‐score (LAZ) at birth was −2.6, −3.9, −0.6 and 0.5 for Groups 1–4, respectively. Although both Groups 1 and 2 were stunted at birth, stunting persisted in Group 1 while children in Group 2 recovered by the fourth month. Group 3 exhibited mild stunting while Group 4 was normal. Wasting and underweight were observed in all groups, with the highest prevalence of underweight in Group 1. Wasting gradually increased among children born already stunted (Groups 1 and 2). This showed the importance of distinguishing children by their growth patterns rather than aggregating them and only comparing population averages against global growth standards. The design of nutrition interventions should consider the differential factors and potential for growth gains relative to different risks within each group.

## Key messages

- In a sample of Ugandan infants, the onset of stunting occurred in utero and set in motion a range of possible growth trajectories for the child after birth.
- Child growth can be characterised by diverse patterns that suggest temporal variations in children's growth. This provides an opportunity for policymakers to provide specialised and targeted nutrition interventions.
- Addressing low birth weight, preterm births and small‐for‐gestational‐age is critical to effectively reduce rates of stunting in rural Uganda.

## Graphical abstract

Growth (length‐for‐age trajectories) in rural Ugandan children can be described in four distinct patterns or groups; the chronically stunted group, the recovery group, the borderline stunted and the normal group.

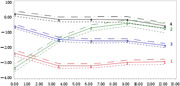

## 1 INTRODUCTION

The World Health Organization (WHO) defines stunting in children under 5 years as a length/height for age *z*‐score less than minus 2 standard deviations (SDs) below the median growth standard established for children globally (World Health Organization, 2017). In 2020, an estimated 149 million preschoolers were stunted worldwide (United Nations Children's Fund UNICEF World Health Organization and International Bank for Reconstruction and Development/The World Bank, 2021). Despite mitigation and strategic efforts, Africa is still faced with unacceptably high numbers of stunted children. In Uganda, for instance, the prevalence of stunting among preschoolers in 2020 was estimated to be almost 28% (World Health Organization and United Nations Children's Fund [UNICEF], 2021).

Numerous studies have been conducted to understand the prevalence, pathogenesis and associated risk factors for stunted growth (Kikafunda et al., 2014; Lauer et al., 2019; Owino et al., 2016; Ssentongo et al., 2020). However, most of these have been observational in nature. The majority of these studies have the benefit of being more resource‐efficient and time‐saving compared to longitudinal studies, but they cannot be used to determine sequences of events or identify patterns over time. One of the few longitudinal (intergenerational) studies was undertaken by the Institute of Central America and Panama (INCAP; Martorell et al., 1995) and was conducted between 1969 and 1977 in rural Guatemala. The aim was to assess the lasting nutrition impact of providing a high‐protein supplement (*Incaparina)* to infants and young children in Central America. The study's legacy, which also demonstrated the value of longitudinal designs, is rooted in follow‐up surveys that traced index participants several decades later (Martorell et al., 1995). A more recent example that harnessed the power of longitudinal approaches was the Young Lives Study (YL; Boyden, 2016) conducted in Ethiopia, India, Peru and Vietnam, which followed 12,000 children over 15 years to determine lasting nutrition impacts on early health insults and poverty (Cueto et al., 2020; Favara & Hoeffler, 2020; Portela & Atherton, 2020). Although informative, these kinds of studies focused on the analyses of means for specific outcomes of the population under study but did not determine if a child would have more, low or no length gains compared to other children of the same age at the same point in time.

In this study, we argued that rates of growth and impacts of associated risk factors are not homogenous at each time point when a young child is measured. The approach used here (group‐based trajectory modelling; Nagin, 1999), allows us to build on the strengths of longitudinal designs by analysing how an outcome evolves over time. We estimated the proportion of the sample that was chronically stunted from birth, exploring why some children recovered while others remained stunted and revealed the differential effect of associated risk factors even among children with the same nutrition outcome. This analytic approach also provided more context about the coexistence of other forms of undernutrition over time.

Group‐based trajectory models categorise individuals with similar attributes into distinct meaningful subgroups (Nagin, 1999). They have been used in various disciplines to identify important patterns within populations. Recent applications include the assessment of psychological disorders in children, academic motivation in school, depressive symptoms in older populations, and crime and the risk of recidivism (Anderson et al., 2010; Galera et al., 2011; Hsu, 2012; Ratelle et al., 2004). In clinical and social sciences research, group‐based trajectory models have been critical in understanding developmental trajectories. For instance, the body mass index (BMI) of children from birth to 3 years of age has been shown to follow four discrete patterns over time: low, intermediate, high and accelerating growth groups, in order of increasing standardised BMI at birth. Children born to mothers who were obese during early pregnancy were more likely have an accelerating growth trajectory compared to the intermediate category (Carter et al., 2012; Giles et al., 2015; Pryor, 2011; Ziyab et al., 2014). In this study, we applied the group‐based trajectory approach to a cohort of children's length‐for‐age *z*‐scores from birth through 1 year of age and explored how trajectories contextualised the coexistence of stunting with underweight and wasting. The specific aim was to identify discrete growth patterns in Ugandan infants and to assess the pre‐ and post‐natal factors associated with each pattern.

## 2 METHODS

### 2.1 Study design and data collection

This study was based on a cohort of infants (*n* = 4528) included in the Uganda Birth Cohort study (UBC; NCT04233944). The UBC was a prospective longitudinal study conducted between 2014 and 2016; it followed pregnant women and their infants every 3 months until the child was 1 year old (Bater et al., 2020; Madzorera et al., 2021). Village health teams (VHTs) recruited 5044 pregnant women in 16 sub‐counties in 8 districts of Northern and Southwestern Uganda. Maternal, child and household characteristics were collected during pregnancy, at birth, and 3, 6, 9 and 12 months from the date of delivery. Serum samples for a subset of mothers at birth and their infants at 6 months of age were collected to assess aflatoxin B1‐lysine levels, iron and vitamin A biomarker concentrations and inflammation. These were processed at VitMin Lab in Germany for α‐1 acid glycoprotein (AGP), C‐reactive protein (CRP), ferritin, soluble transferrin receptor (sTfR) and RBP levels. The Peanut and Mycotoxin Innovation Lab at the University of Georgia processed serum samples for aflatoxin B1‐lysine adducts (AFB1) using the high‐performance liquid chromatography (HPLC)‐fluorescence method (Qian et al., 2013).

At each study visit, three repeated measurements of weight and length were obtained for each child, using a Seca scale and Shorr board, respectively. Length‐for‐age *z*‐scores (LAZ) were calculated using WHO standards (World Health Organization, 2006). Extreme LAZ values were defined as LAZ <−6 or >6 and excluded from analyses. The Higher Degrees, Research and Ethics Committee (HDREC) of Makerere University School of Public Health, the Institutional Review Board (IRB) from Tufts Health Sciences and Harvard T. H. Chan School of Public Health approved this study. All participants provided written consent before the study began.

### 2.2 Statistical analysis

#### 2.2.1 Identifying implausible length values over time

We assessed children's length values for outliers. Length values were within plausible ranges at each time point, but not always across time. We, therefore, used conditional growth percentiles (Yang & Hutcheon, 2016), together with a supplementary rudimentary algorithm in which we excluded observations with the same length values at any time point from birth to 6 months, or observations with an arbitrary length difference of 20 cm at the subsequent visit. Conditional growth percentiles, in this context, were calculated based on the child's previous length. Briefly, a random‐effects model was used to estimate population average lengths and their corresponding variation (Yang & Hutcheon, 2016). These estimates were then used to calculate a length value and SD range, which accounted for the previous length. A range of ±4 SD was used initially, but this range was found to be insufficiently precise to capture implausible lengths. Thus, a smaller range of ±3 SD was used to reveal more outliers in the data. This cut‐off is supported by the rule‐of‐thumb for determining outlying values in normally distributed data (Dunn, n.d.).

#### 2.2.2 Group‐based trajectory modelling

We used the *TRAJ* procedure in SAS to run an unconditional (only time is accounted for) group‐based trajectory model (Nagin, 1999). To determine the optimal number of trajectory groups, we compared Bayesian information criteria (BIC) values for up to six trajectory groups and chose the model with the least negative value. In addition, we looked for a statistically significant quadratic trajectory and reasonable interpretation (based on a priori knowledge of growth trends) of length trajectory shapes (World Health Organization, 2006), to avoid redundancy in groups. We also set a minimum group membership probability of no less than 5%, that is, the final model had at least 5% of the total sample size of children. An additional aspect of this model is its use of polynomial terms to account for the complex changes in LAZ over time, as seen in Equation (1) below.

(1) $$Y_{it}^{*j}=\beta_{0}^{j}+\beta_{1}^{j}.Age_{it}+\beta_{2}^{j}.Age_{it}^{2}+\beta_{3}^{j}.Age_{it}^{3}+\varepsilon_{it}\text{…}.$$

*Y* ^*j^_*it*_ is the predicted LAZ for subject *i* at time *t*, given membership in group *j. Age*_*it*_ is the child's age at a time *t*. It was squared and cubed to represent significant higher‐order polynomials. The beta estimates, *β* ^*j*^_0_, *β* ^*j*^_1_, *β* ^*j*^_2_ and *β* ^*j*^_3_ determined the nature of the trajectories and they can vary across groups. *Ԑ* is a normally distributed error with a mean of zero and constant variance.

To determine the degree of polynomial terms, we tested models up to the cubic trajectory for statistical significance and selected the four‐trajectory group model with the order 3 2 3 3. The number of digits corresponds to the number of groups in the model while the value refers to the order of the polynomial; 1 is linear, 2 is quadratic and 3 is cubic.

During the model estimation process, each child's probability of belonging to a trajectory group was determined using estimates called posterior probabilities. The maximum‐probability assignment rule was used to allocate a specified trajectory group to each child. Thus, the group with the highest probability determined the final group assignment for each child. As a robustness check, we used these probabilities to assess whether the trajectories obtained effectively identified children with similar LAZ scores. If the mean posterior probabilities for children in each group were above a cut‐off value of 0.7 (Andruff et al., 2009), then the trajectories were considered highly reliable.

A multinomial regression model was conducted to understand the relationship between risk factors and the trajectory groups obtained from the unconditional model, by comparing differences across groups. Risk factors included maternal education, height, age, distance to a water source, food insecurity (Coates et al., 2007), exclusive breastfeeding and child's diet diversity (World Health Organization, 2008), preterm birth (gestational age < 37 weeks) and birth weight. Posterior probabilities obtained from the multinomial logit model were averaged across children's characteristics to create a descriptive profile. We also conducted a sub‐analysis using smaller sample sizes to assess the effects of human immunodeficiency virus (HIV) status (*n* = 2548), maternal aflatoxin exposure (*n* = 3092) and maternal iron (*n* = 1578) and maternal vitamin A status (n = 1578) on group membership. All confounders were adjusted based on literature reviews and a priori knowledge (Ren et al., 2021; Wamani et al., 2004). For example, maternal aflatoxin has been shown to negatively affect growth in Ugandan children (Lauer et al., 2019). Evidence of the deleterious effects of maternal HIV (Magadi, 2011) and micronutrient deficiencies in pregnancy (Iftikhar et al., 2018) on growth have also been established. All analyses were performed using SAS v 9.4.

## 3 RESULTS

### 3.1 Implausible length values

Figure S1 demonstrates the conditional growth percentiles strategy to identify implausible lengths. We identified and excluded 22 observations based on this method and an additional 255 observations using the rudimentary approach. The final sample size resulted in 15,836 data points across time, which translated into a loss of 2% of the data. The greatest reduction in sample size was observed at the 6‐month time point (3%). A proportion of 10% or more is likely to bias estimates (Bennett, 2001) so our proportion of missing data likely had minimal effects on the results.

### 3.2 Group‐based trajectories

**Figure 1:**
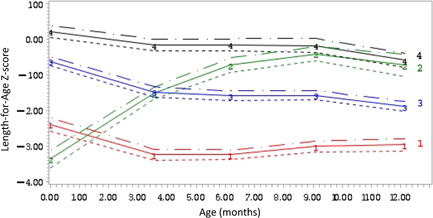
Four distinct LAZ trajectories identified in rural Ugandan infants from birth to 12 months of age. Group 1 is the chronically stunted group (18% of the children), Group 2 is the recovery group (10%), Group 3 is the borderline stunted group (51%) and Group 4 is the normal group (21%). Dashed lines represent confidence intervals. LAZ, length‐for‐age *z*‐score

Figure 1 shows the four distinct trajectory groups that were identified in this sample of Ugandan children: a chronically stunted group (Group 1), a recovery group (Group 2), a borderline stunted group (Group 3) and a normal group (Group 4). In Group 1, infants were born stunted (LAZ < −2 SD) and they remained stunted at 12 months of age. In Group 2, children were born stunted (LAZ < −2 SD), after which their LAZ improved significantly around the 3‐month time point; after that, they remained in the ‘normal’ range (LAZ > −2 SD). Infants in Group 3 were mildly stunted (LAZ close to −1 SD) at birth and their LAZ scores gradually declined to come very close to the stunting cut‐off of LAZ < −2 SD. The latter were categorised as borderline stunted. Infants in Group 4 were born with LAZ scores above 0, stayed above 0 for most of the year, but gradually declined to start at 9 months of age. The proportion of children in Groups 1, 2, 3 and 4 were 18%, 10%, 51% and 21%, respectively.

### 3.3 Descriptive statistics

In Table 1, we present maternal, household and infant characteristics, stratified by trajectory groups. The average age of the mother at the child's birth was 27 years. We observed significant group differences in age; the youngest mothers had the highest proportion of children who belonged to Group 2. The average maternal height was 159 cm, with less than 1% of them being of short stature (<145 cm; Ozaltin et al., 2010; Subramanian et al., 2009). Educated mothers, mothers from a high socioeconomic status, or those who met their own minimum diet diversity, had the highest proportion of children in Group 4 relative to other groups. Of all the mothers, 7% were HIV‐positive, 9.2% had children in the recovery group (Group 2), followed by 8.6% with children in the chronically stunted group (Group 1). Mothers of children in Group 2 had the highest median maternal aflatoxin levels.

**Table 1:** Maternal, household and infant characteristics by group membership

	Total	Group 1 chronically stunted, *N* a = 731	Group 2 recovery group, *N* = 359	Group 3 borderline stunted, *N* = 2657	Group 4 normal group, *N* = 781
	*n* (%)	*n* (%)	*n* (%)	*n* (%)	*n* (%)
*Maternal characteristics*					
**Education in years (*N*** = **4403)**					
<6	2141 (48.6)	445 (62.0)	154 (43.4)	1234 (47.9)	308 (40.7)
6–11	2147 (48.8)	264 (36.8)	194 (54.6)	1271 (49.4)	418 (55.3)
12+	115 (2.6)	9 (1.2)	7 (2.0)	69 (2.7)	30 (4.0)
**Age in years (*N*** = **4120)**					
16–20	663 (16.1)	117 (17.3)	77 (22.6)	383 (16.1)	86 (12.1)
21–24	1116 (27.1)	170 (25.2)	99 (29.0)	653 (27.3)	194 (27.2)
25–29	1062 (25.8)	168 (24.9)	74 (21.7)	623 (26.1)	197 (27.6)
30–34	756 (18.4)	117 (17.3)	63 (18.5)	434 (18.2)	142 (19.9)
≥35	523 (12.7)	103 (15.3)	28 (8.2)	298 (12.5)	94 (13.2)
**Height in cm (*N*** = **4278)**					
<145	30 (1.0)	15 (2.2)	0 (0.0)	13 (0.5)	2 (0.3)
145–149.9	259 (6.1)	70 (10.0)	23 (6.5)	153 (6.1)	13 (1.8)
150–154.9	840 (19.6)	168 (24.1)	72 (20.2)	500 (20.0)	100 (13.8)
155–159.9	1318 (30.8)	216 (31.0)	116 (32.6)	780 (31.2)	206 (28.4)
>160	1831 (42.8)	229 (32.8)	145 (40.7)	1052 (42.2)	405 (55.8)
**MDDW at birth (*N*** = **4361)** b	540 (12.4)	56 (7.9)	52 (14.5)	323 (12.7)	109 (14.7)
**Anaemia (*N*** = **3873)** c	561 (14.5)	95 (14.7)	49 (15.0)	332 (14.8)	85 (13.0)
**Iron deficiency (Ferritin; *N*** = **1578)** c	198 (12.6)	31 (13.0)	17 (12.9)	115 (12.3)	35 (12.9)
**Iron‐deficient erythropoiesis (*N*** = **1578)** c	339 (21.5)	50 (20.9)	19 (14.4)	215 (23.0)	55 (20.3)
**Inflammation (*N*** = **1578)** c	872 (55.3)	141 (59.0)	72 (54.6)	521 (55.7)	138 (50.9)
**HIV‐positive mothers (*N*** = **2548)**	169 (6.6)	33 (8.6)	20 (9.2)	92 (6.3)	24 (5.0)
**Median aflatoxin B1‐lysine adduct levels (*N*** = **3092)**	3.2 (IQR = 7.2)	2.7 (IQR = 6.8)	4.4 (IQR = 8.6)	3.2 (IQR = 7.1)	3.2 (IQR = 6.9)
*Household characteristics*					
**Wealth index (*N*** = **4386)**					
Poorest	874 (19.9)	122 (17.0)	73 (20.6)	543 (21.2)	136 (18.1)
Poorer	861 (19.6)	174 (24.3)	72 (20.3)	505 (19.7)	110 (14.6)
Middle	882 (20.1)	202 (28.2)	66 (18.6)	495 (19.3)	119 (15.8)
Richer	887 (20.2)	124 (17.3)	73 (20.6)	506 (19.7)	184 (24.5)
Richest	882 (20.1)	94 (13.1)	70 (19.8)	515 (20.1)	203 (27.0)
**HFIAS (*N*** = **4399)**					
Food secure	1681 (38.2)	230 (32.0)	172 (48.5)	961 (37.4)	318 (42.1)
Mildly food insecure access	1092 (24.8)	175 (24.4)	71 (20.0)	647 (25.2)	199 (26.3)
Moderately food insecure access	1019 (23.2)	186 (26.0)	71 (20.0)	597 (23.2)	165 (21.8)
Severely food insecure access	607 (13.8)	127 (17.7)	41 (11.6)	365 (14.2)	74 (9.8)
**Southwest region (*N* = 4403)**	2192 (49.8)	370 (51.5)	161 (45.4)	1239 (48.1)	422 (55.8)
*Infant characteristics*					
**Female child (*N* = 4245)**	2139 (50.4)	266 (38.1)	195 (54.6)	1233 (50.0)	445 (61.3)
**Exclusive breastfeeding (birth; *N* = 4301)**	3915 (91.0)	614 (87.6)	334 (93.4)	2306 (91.8)	661 (90.4)
**Exclusive breastfed at 3 months (*N* = 3558)**	3558 (100)	565 (100)	274 (100)	2095 (100)	624 (100)
**Low birth weight (*N* = 4238)**	186 (4.4)	67 (9.6)	25 (7.0)	90 (3.7)	4 (0.6)
**Preterm (*N* = 3902)**	849 (21.8)	178 (27.3)	85 (26.2)	494 (21.7)	92 (14.1)
**Child met MDDS (6 months; *N* = 3162)**	129 (4.1)	17 (3.1)	11 (4.6)	67 (3.7)	34 (5.6)
**Child met MDDS (9 months; *N* = 2930)**	92 (3.1)	12 (2.4)	8 (3.6)	46 (2.8)	26 (4.7)
**Child met MDDS (12 months; *N* = 2077)**	198 (9.5)	31 (8.9)	18 (10.7)	114 (9.8)	35 (8.8)

*Note*: HFIAS, Household Food Insecurity and Access Scale (Coates et al., 2007); MDDS, Minimum Diet Diversity score among 6‐ to 23‐month‐old children (at least four of the seven food groups based on the Infant and Young child‐feeding practices; World Health Organization, 2008).

^a^ Maximum frequency in each group. There are variations in sample sizes for each risk factor due to missing values or subgroups for micronutrient variables and HIV sero‐status. Percentage estimates are column percentages for each group. Denominators used have not been presented.

^b^ Women's Minimum Diet Diversity score (≥5 food groups; FAO and FHI 360, 2016).

^c^ Anaemia was defined as Hgb < 11 g/dl, iron deficiency defined as Fer < 15 µg/L and iron‐deficient erythropoiesis defined as sTFR > 8.3 mg/L, vitamin A deficiency is defined as (RBP < 0.7 µmol/L). Inflammation was defined as CRP ≤ 5 or AGP > 1.

**Figure 2:**
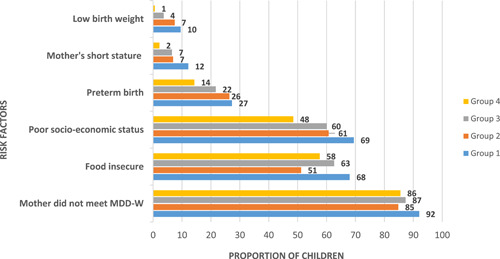
Differences in key risk factors across growth trajectories/groups. Low birth weight was defined as birth weight < 250 g. Mother's short stature was defined as maternal height < 145 cm. Preterm births were defined as gestational age < 37 weeks. Poor households were defined as households in the poorest, poor and middle categories of the Demographic Health Survey's asset‐based classification of wealth. Food insecurity includes mild, moderate and severe food insecurity access. Mothers’ diet diversity score (MDD‐W) was based on consumption of five food groups or more

Regarding infant characteristics, the highest proportion of girls belonged to Group 4, the normal group, while the chronically stunted group, Group 1, had the highest proportion of infants born preterm or with low birth weight. Differences in infant characteristics across groups are presented in Figure 2. In contrast to other groups, Group 1 had the highest proportion of children from the poorest and most food‐insecure households. This group had the greatest number of preterm births and infants with low birth weight. Their mothers had less diverse diets and were shorter than those in all other groups.

### 3.4 Posterior probabilities

The average posterior probabilities for each group in sequence were 0.78, 0.72, 0.74 and 0.74, respectively, indicating high reliability. Table S1 shows posterior probabilities for the multinomial logit regression model. Compared to the model with no risk factors, children in the recovery group were more likely to be male, born preterm, and have low birth weight. Mothers of children from both groups in which children were born stunted (Groups 1 and 2) were more likely to be iron deficient and come from poor households. Maternal aflatoxin exposure was not associated with an increased risk of membership to these two groups; however, coupled with poor socioeconomic status, we observed a 2% increase in the risk of belonging to the recovery group (Group 2). These probabilities, while informative, were only univariate assessments of the association between risk factors and group assignment.

### 3.5 Multinomial logit regression

Regression estimates from the multinomial logit model are presented in Table 2. The risk factors that increased the likelihood of membership to either the chronically stunted or recovery groups were; increased household distance to a water source (*β* = 0.14 *p* = 0.041), being a poor household (Group 1; *β* = 0.80, *p* < 0.001, Group 2; *β* = 1.85, *p* < 0.001) and being preterm at birth (Group 1; *β* = 0.41, *p* = 0.037, Group 2; *β* = 0.81, *p* < 0.001). Although children in both groups were stunted at birth, the magnitude of effects associated with measured risk factors was different by group. For instance, in comparison to the normal group, children from low socioeconomic households had a twofold increase in the likelihood of Group 2 membership vis‐à‐vis Group 1 and increased maternal education and height were more protective of Group 2 membership. Interestingly, these two groups also had children who were more likely to be exclusively breastfed (Group 1; *β* = 2.81, *p* < 0.001, Group 2; *β* = 1.89, *p* < 0.001).

**Table 2:** Multinomial logit regression estimates predicting group assignment

	Group 1		Group 2		Group 3	
	Chronically stunted		Recovery group		Borderline stunted	
	Estimate	95% CI	Estimate	95% CI	Estimate	95% CI
Mother's education (years)	−0.167	−0.334 to −0.000	−0.503	−0.681 to −0.325***	−0.12	−0.255 to 0.015
Mother's height (cm)	−0.43	−0.595 to −0.265***	−0.9	−1.067 to −0.733***	−0.417	−0.55 to −0.284***
Mother's age (years)	−0.183	−0.344 to −0.022*	−0.052	−0.205 to 0.101	0.018	−0.109 to 0.145
Mother's MDD‐W at birth	0.174	−0.269 to 0.617	−0.471	−0.977 to 0.035	−0.14	−0.542 to 0.262
Distance to water (km)	0.142	0.005–0.279*	0.01	−0.145 to 0.165	0.054	−0.075 to 0.183
Infant's gender (female)	0.088	−0.222 to 0.398	−0.859	−1.161 to −0.557***	−0.63	−0.891 to −0.369***
Food insecurity^a^	0.183	−0.129 to 0.495	−0.107	−0.426 to 0.212	−0.354	−0.623 to −0.085**
Poor household^b^	0.802	0.410–1.194***	1.854	1.468–2.24***	0.55	0.225–0.875***
Low birth weight (kg)	4.786	−8.914 to 18.486	5.585	−8.08 to 19.25	3.664	−10.205 to 17.533
Exclusive breastfed at birth	2.812	2.204–3.420***	1.889	1.432–2.346***	0.165	−0.268 to 0.598
Diverse diets (6 months)^c^	0.149	−0.009 to 0.308	−0.249	−0.416 to −0.082**	0.026	−0.105 to 0.157
Preterm birth^d^	0.405	0.025–0.785*	0.814	0.455–1.173***	0.159	−0.176 to 0.494
Northern region	0.191	−0.215 to 0.596	−0.483	−0.873 to −0.093**	0.038	−0.305 to 0.381
Constant	−3.892	−4.462 to −3.322***	−2.9	−3.329 to −2.471***	0.421	−0.104 to 0.946
Mother's HIV‐positive status^e^	1.197	0.358–2.036**	1.391	0.589–2.193**	0.757	0.02–1.494*
Mother's AFB1 levels	0.177	−0.035 to 0.389	0.187	−0.025 to 0.399	0.104	−0.084 to 0.292
Mother is iron deficient (ferritin)^f^	−22.682	>10	−0.314	−0.882 to 0.254	0.137	−0.375 to 0.649
Iron‐deficient erythropoiesis^f^	−0.008	−0.523 to 0.507	0.079	−0.448 to 0.606	−0.209	−0.67 to 0.252
Mother is Vitamin A deficient^f^	−0.005	−2.204 to 2.19	0.926	−1.114 to 2.966	−0.185	−2.198 to 1.828

*Note*: Reference is Group 4 (normal LAZ scores).

^a^ Food insecurity includes mild, moderate and severe food insecurity access (Coates et al., 2007).

^b^ Poor households were defined as households in the poorest, poor and middle categories of the Demographic Health Survey's asset‐based classification of wealth.

^c^ Diverse diets at 6 months of age are defined as consumption of at least four of the seven defined food groups in the Infant Young and Child Feeding Practices indicators (World Health Organization, 2008).

^d^ Preterm was defined as gestational age < 37 weeks.

^e^ HIV status and biomarker estimates are sub‐analyses and separate models from the main model. Maternal aflatoxin, Ferritin, sTFR and RBP have been adjusted for inflammation (CRP and AGP).

^f^ Anaemia was defined as Hgb < 11 g/dl, iron deficiency defined as Fer < 15 µg/L and iron‐deficient erythropoiesis defined as sTFR > 8.3 mg/L, Inflammation was defined as CRP ≤ 5 or AGP > 1. Vitamin A deficiency is defined as (RBP < 0.7 µmol/L). BRINDA adjustments for inflammation were calculated for Ferrin, sTFR and Vitamin A (Larson et al., 2018; Namaste et al., 2017; Rohner et al., 2017).

* *p *≤ 0.05.

** *p *≤ 0.01.

*** *p *≤ 0.001.

### 3.6 Concurrence of underweight and wasting

**Figure 3:**
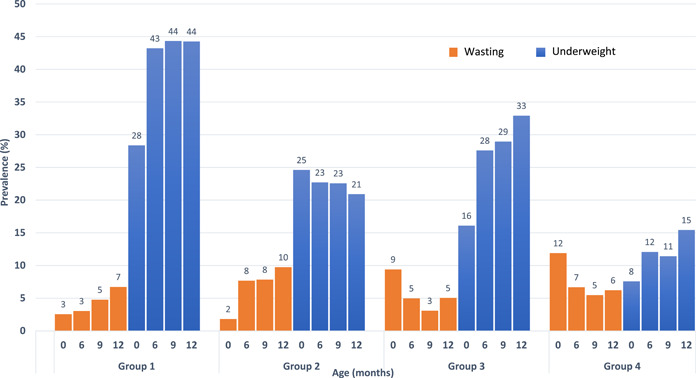
Coexistence of wasting and underweight over time by LAZ trajectory groups. Group 1 is the chronically stunted group, Group 2 is the recovery group, Group 3 is the borderline stunted group and Group 4 is the normal group. LAZ, length‐for‐age *z*‐score

Figure 3 shows the variability in the coexistence of underweight (weight‐for‐age < −2) and wasting (weight‐for‐height < −2) in children up to 1 year of age. Underweight was a major problem in all four trajectory groups, with alarming rates (prevalence > 40%) among children who were chronically stunted (Group 1). This group also faced an upward trend in the prevalence of wasting. Although children in Group 2 recovered from stunting, they were burdened with an increasing prevalence of wasting and relatively high rates of underweight compared to Group 4 with normal LAZ scores (LAZ > −2). The prevalence of wasting at birth was highest among children with normal LAZ scores.

In the sub‐analyses, we found that HIV‐positive mothers were significantly associated with a 119% increase in the likelihood of having children who belonged to the chronically stunted group (Group 1), and a 139% increase in the likelihood of belonging to the recovery group (Group 2). Maternal iron and vitamin A deficiency were not associated with membership in any of the groups.

## 4 DISCUSSION

Our findings show that four distinct trajectory groups usefully described children's growth patterns in this sample of rural Ugandan children from birth to 12 months of age. At birth, each group started at different points on a length scale and subsequently followed a separate trajectory, with no overlap except for the recovery group, which crossed over from stunting levels to normal LAZ levels, ultimately surpassing the borderline stunted group. These findings are similar to those found in a multi‐country study (Young Lives), which took a life‐course approach in assessing malnutrition from 1 to 15 years for younger children and 8 to 22 years for older children in Ethiopia, India, Peru and Vietnam (Schott et al., 2019). The authors identified three trajectories and named them low, medium and high, to intuitively symbolise levels of stunting. They found that maternal education, wealth, and urban residence were associated with a lower risk of being in the high or medium stunting groups.

The categorisation of length‐for‐age *z*‐scores before birth has been observed in other studies (de Onis & Branca, 2016; Prendergast & Humphrey, 2014), suggesting a significant influence of prenatal risk factors. After parturition, environmental influences may lead to significant changes in LAZ over time at the individual level. Infants with similar LAZ shifts can then be grouped, as demonstrated in this study.

At the group level, these effects either reinforce membership to prenatally assigned groups, or promote shifts in populations across groups. We have shown that poverty, preterm birth and increased distance to a water source increase the likelihood of belonging to the groups in which children were stunted at birth, which is in line with other studies (Budhathoki et al., 2020; Mawa & Lawoko, 2018). Regarding maternal factors, the most differentiating were higher education, maternal height and mothers’ age, with each being protective of stunting at birth. While research has shown that older maternal age is associated with an increased likelihood of stunting (Esfarjani et al., 2013), we found no significant increase in stunting for children whose mothers were aged 35 years and older.

In the recovery group (Group 2), the shift from stunting to normal LAZ was observed at about the 3‐month time point. It is unclear why these infants recovered; one possible reason is that this group could have received special medical attention, considering that they were born severely stunted. The surprising finding that exclusive breastfeeding was more likely to be associated with stunting at birth (relative to the normal group) may be explained by increased maternal care of babies who were clearly not thriving (i.e., reverse causality; Marquis et al., 1997). Mothers of infants who are visibly malnourished, as is the case for infants who appear smaller or shorter than expected, are more likely to breastfeed them to achieve the remedial benefits of breastmilk. These mothers are also more likely to receive medical advice than those with seemingly healthy children (Bwalya et al., 2017).

In all four trajectory groups, we observed the coexistence of underweight and wasting from birth through the first year of age at varying levels within each group over time. This concurrence has been observed in other studies conducted in various countries, including Uganda (Khara et al., 2017; Kundan et al., 2021; Myatt et al., 2018; Odei et al., 2020). The widely perceived notion that defines wasting as acute malnutrition and stunting as chronic (Caulfield et al., 2006; Dipasquale et al., 2020; Lenters et al., 2016) is contrary to our findings. Compared to other groups, coexistence with underweight was much more pronounced in the chronically stunted group (Group 1), and the prevalence of wasting increased gradually over time. That a substantial portion of sampled children (18%; *n* = 731) was faced with this triple burden (wasting, underweight and stunting) is concerning. Concurrence has been linked to an increase in mortality risk compared to affliction with a single burden (McDonald et al., 2013; Wright et al., 2021), and being wasted can in turn lead to stunting (Briend et al., 2018; Childhood Infection and Malnutrition Network, 2012). Moreover, the current intervention strategies that recognise and address concurrence are still scanty. These reasons, coupled with the finding that wasting and underweight are prevalent even among the group with normal LAZ scores (Group 4), furthermore, emphasise the importance of a comprehensive assessment of malnutrition in all of its forms at the individual level and highlight the urgent need for targeted and specialised interventions.

Discrete trajectories characterising children's growth over time suggest that while all children are responsive to nutrition interventions, a policy and programme focus should be on prioritising those interventions that encompass all forms of malnutrition and are most likely to address the child's needs based on mortality risk level and the expected or probable linear growth trend. Current effective strategies that reduce stunting have been grouped into nutrition‐specific interventions that address immediate causes and nutrition‐sensitive for the underlying determinants (Black et al., 2013). Bhutta et al. (2020) proposed reclassifying them into direct and indirect approaches within the health sector and supportive approaches within non‐health sectors, such that direct approaches are synonymous with nutrition‐specific interventions and indirect approaches with nutrition‐sensitive, except they are sub‐stratified within health and non‐health sectors. We suggest that the implementation of these strategies if combined with the knowledge of a child's probable linear growth trajectory given their current nutrition status, will not only address the complex underlying causal dynamics and prioritise children at the highest risk of adverse outcomes or death, but also seemingly self‐correct malnutrition at scale. For instance, for children who are stunted at birth, coexistence or development of wasting and/or being underweight are likely threats, so nutrient delivery together with medical interventions (direct approaches within the health sector) may effectively prevent the likely trajectory observed in Group 1, the chronically stunted group. For children born with normal LAZ ranges, strategies such as those geared towards improving livelihoods, and increasing maternal education and awareness (indirect or supportive approaches within the health and non‐health sector), are more likely to work well to prevent the risk of trending towards stunting as observed in Group 3. This recommendation is in line with other policy recommendations that advocate for targeted interventions (Wells et al., 2019).

One of the limitations of this study was our use of the 2008 definition of children's minimum dietary diversity (World Health Organization, 2008), instead of the current definition (World Health Organization and United Nations Children's Fund (UNICEF), 2021), which incorporates breast milk intake in five out of eight food groups. The definition used in these analyses was based on the consumption of at least four out of seven food groups during the previous day, excluding breast milk. Our results should therefore be interpreted with this in mind.

Another limitation of this study is the relatively small sample size available for the sub‐study. We looked at the effects of dietary aflatoxin, micronutrient status and HIV serostatus on maternal and child outcomes. We found that HIV‐positive mothers had a higher likelihood of producing stunted children relative to the normal group, which has been established in other studies conducted in East Africa (Abate et al., 2020; McGrath et al., 2012).

## 5 CONCLUSION

Child linear growth followed four distinct trajectories in this sample from Uganda, making it important to distinguish where children fall in this typology rather than lumping them together and only comparing against global growth standards. The magnitude of the effect of risk factors and concurrence with other nutrition outcomes varied across groups, which suggests that the design of nutrition interventions should at least be better informed of the differential factors and potential for growth gains relative to risks within each group. The onset of stunting occurred before birth, with maternal education, maternal height, maternal age, HIV serostatus and gestational age as some of the possible underlying prenatal factors.

## AUTHOR CONTRIBUTIONS

Patrick Webb and Shibani Ghosh were the primary contributors to the conception and design. Bernard Bashaasha, Nassul Kabunga and Edgar Agaba provided great support in the data collection, implementation and operation of the project. Grace Namirembe performed the statistical analyses, writing the first draft and consulted with Patrick Webb, Shibani Ghosh and Lynne M. Ausman. Patrick Webb, Shibani Ghosh, Lynne M. Ausman, Sonia Zaharia, Robin Shrestha and Julieta Mezzano edited the manuscript and provided intellectual content. All authors have read and approved the final manuscript.

## CONFLICTS OF INTEREST

The authors declare no conflicts of interest.

## ETHICS STATEMENT

The study was conducted according to the World Medical Association Declaration of Helsinki and was approved by the ethics committees of the Institutional Review Boards of Tufts University. Written informed consent was obtained from all participants before their participation in the study.

## Supporting information

Supporting information.

Supporting information.

## Data Availability

The data that support the findings of this study are available from the corresponding author upon reasonable request.
