# Supplementary figures and images for: Child stunting starts in utero: Growth trajectories and determinants in Ugandan infants
Source: Matern Child Nutr. 2022 Apr 29;18(3):e13359. doi: 10.1111/mcn.13359 (PMC9218325; doi:10.1111/mcn.13359)

*
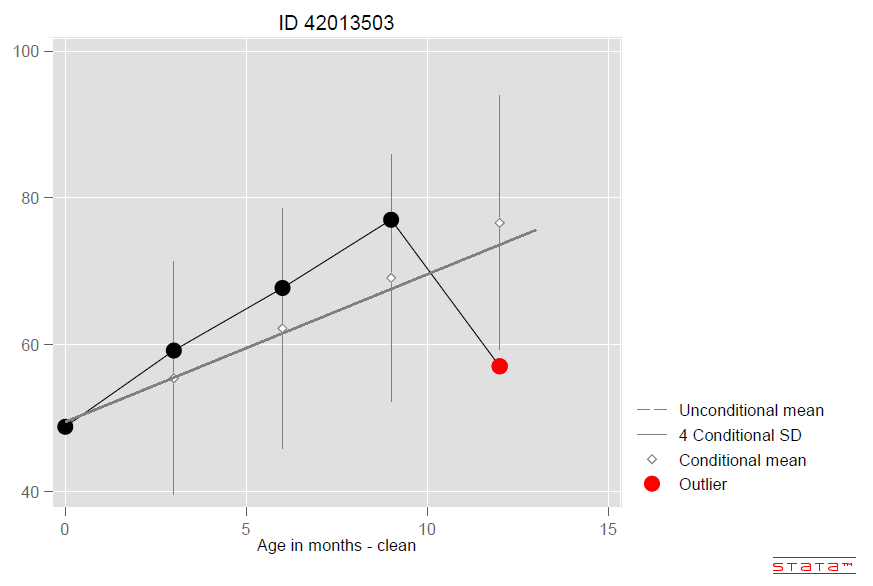
*

**Figure 1S**. Outlying height value obtained using the conditional growth percentile method

Supplement: Supplementary file 1 — Supporting information. [file MCN-18-e13359-s002.docx]
