# Supplementary material for: Child stunting starts in utero: Growth trajectories and determinants in Ugandan infants
Source: Matern Child Nutr. 2022 Apr 29;18(3):e13359. doi: 10.1111/mcn.13359 (PMC9218325; doi:10.1111/mcn.13359)
Supplement: Supplementary file 2 — Supporting information. [file MCN-18-e13359-s001.docx]

**Supplementary information**

**Table 1S.** Posterior probabilities showing the effect of select risk factors on group membership.

|  | **Group 1**  **Chronically stunted** | **Group 2**  **Recovery group** | **Group 3**  **Borderline stunted** | **Group 4**  **Normal group** | **N** |
| --- | --- | --- | --- | --- | --- |
| **No risk factors** | 0.09 (0.08,0.10) | 0.11 (0.10,0.12) | 0.48 (0.47,0.49) | 0.32 (0.30,0.33) | 2522 |
| **Male gender** | 0.08 (0.07,0.10) | 0.12 (0.11,0.14) | 0.47 (0.45,0.49) | 0.32 (0.31,0.34) | 2106 |
| **Preterm^a^** | 0.10(0.08,0.12) | 0.13(0.10,0.15) | 0.48(0.45,0.51) | 0.30(0.27,0.32) | 849 |
| **Low Birth weight^b^** | 0.08(0.03,0.13) | 0.13(0.07,0.19) | 0.48(0.41,0.54) | 0.31(0.25,0.37) | 186 |
| **Poor SES^c^** | 0.11(0.10,0.13) | 0.14(0.12,0.15) | 0.45(0.43,0.47) | 0.30(0.28,0.32) | 2617 |
| **Maternal iron deficiency^d^** | 0.16(0.12,0.20) | 0.11(0.08,0.14) | 0.51(0.46,0.55) | 0.22(0.18,0.26) | 198 |
| **High maternal AFB1 exposure^e^** | 0.07(0.05,0.08) | 0.11(0.08,0.13) | 0.49(0.47,0.52) | 0.34(0.31,0.36) | 773 |
| **High maternal AFB1 and Poor SES** | 0.09(0.06,0.12) | 0.13(0.10,0.17) | 0.47(0.43,0.51) | 0.30(0.27,0.34) | 260 |
| **Inflammation in the mother^f^** | 0.08(0.06,0.10) | 0.13(0.10,0.15) | 0.48(0.45,0.51) | 0.31(0.29,0.33) | 872 |
| Posterior probabilities are based on the adjusted multinomial model. Only the risk factors that are associated with an increase in the risk of Group 1 and 2 membership are presented. Figures in brackets are confidence intervals.  a-Gestational age < 37 weeks  b- Birth weight < 250g  c-Socio-economic status based on the Demographic Health Survey’s asset-based classification of wealth (Rutstein and Kiersten 2004)  d-Maternal iron deficiency was defined as Fer < 15 ug/L and adjusted for inflammation using the BRINDA approach (Larson et al. 2018; Namaste et al. 2017; Rohner et al. 2017).  e-Aflatoxin levels greater than the 75th percentile  f-CRP <= 5 or AGP > 1 | | | | | |
